# Identification of the Causative Disease of Intermittent Claudication through Walking Motion Analysis: Feature Analysis and Differentiation

**DOI:** 10.1155/2014/861529

**Published:** 2014-07-07

**Authors:** Tetsuyou Watanabe, Takeshi Yoneyama, Hiroyuki Hayashi, Yasumitsu Toribatake

**Affiliations:** ^1^The School of Mechanical Engineering, Kanazawa University, Kakuma-machi, Kanazawa 920-1192, Japan; ^2^Department of Orthopedic Surgery, Graduate School of Medical Science, Kanazawa University, Japan; ^3^Department of Orthopedic Surgery, Koseiren Takaoka Hospital, Japan

## Abstract

Intermittent claudication is a walking symptom. Patients with intermittent claudication experience lower limb pain after walking for a short time. However, rest relieves the pain and allows the patient to walk again. Unfortunately, this symptom predominantly arises from not 1 but 2 different diseases: LSS (lumber spinal canal stenosis) and PAD (peripheral arterial disease). Patients with LSS can be subdivided by the affected vertebra into 2 main groups: L4 and L5. It is clinically very important to determine whether patients with intermittent claudication suffer from PAD, L4, or L5. This paper presents a novel SVM- (support vector machine-) based methodology for such discrimination/differentiation using minimally required data, simple walking motion data in the sagittal plane. We constructed a simple walking measurement system that is easy to set up and calibrate and suitable for use by nonspecialists in small spaces. We analyzed the obtained gait patterns and derived input parameters for SVM that are also visually detectable and medically meaningful/consistent differentiation features. We present a differentiation methodology utilizing an SVM classifier. Leave-one-out cross-validation of differentiation/classification by this method yielded a total accuracy of 83%.

## 1. Introduction

Intermittent claudication [1] is a walking symptom. Patients with intermittent claudication suffer from lower limb pain after a walking for a short time. However, rest relieves the pain and allows the patient to walk again. Asintermittent claudication involves trouble walking, patients often consult an orthopedic surgeon, and the number of such consultations has recently increased markedly [2]. Unfortunately, intermittent claudication is predominantly caused by not 1 but 2 different diseases: LSS (lumber spinal canal stenosis) and PAD (peripheral arterial disease). Toribatake et al. [1–3] noted that PAD and LSS produce similar symptoms and emphasized the significance of their differential diagnosis. Therefore, it is clinically very important (especially for orthopedic surgeons) to identify the causative disease of intermittent claudication. Among the types of LSS, we focused on the L4 and L5 subtypes, which cause radicular symptoms that are mainly responsible for intermittent claudication in patients with LSS; these subtypes of LSS are difficult to differentiate from PAD [1]. For this disease, L4 and L5 indicate the vertebral level affected by stenosis.

There are 2 categories of examinations for differentiating between these conditions. Some examinations are simple but imprecise and often fail to differentiate the underlying diseases; 2 examples are palpation and observation of standing posture. The other examinations are precise but invasive and expensive; some examples are angiography, myelography, magnetic resonance imaging (MRI), and measurement of the ankle brachial index (ABI) [4]. Furthermore, these examinations require highly skilled professionals and precision instruments. Such complicated and expensive examinations are difficult to conduct at small hospitals and clinics. Furthermore, their high cost is undesirable for the patient. The optimal differentiation method would be an examination that used a minimal number of simple instruments and could be easily performed even by nonspecialists. Notably, the affected parts of the legs differ between the causative diseases and could thus produce kinematical differences in patients' gait patterns.

In this context, this study was performed to develop a new differentiation methodology utilizing minimally required data; simple walking motion data. The key features of this methodology are as follows.

(*1) The Differentiation Is Based on Minimally Required Walking Motion Data*. Aiming at the usage in small hospitals or at home, the differentiation was designed to use only the 2-dimensional gait pattern in the sagittal plane from the simple walking motion analysis. This presented a challenge. Additional advantages of the system are easy set-up and calibration, a short duration of measurement, and usability in a narrow space and a noncontrolled environment by nonspecialists.

(*2) SVM (Support Vector Machine) [5, 6] Classifier-Based Methodology with a High Rate of Accuracy*. There is no research focusing on differentiating between PAD and LSS using gait feature analysis except for our own previous reports [3, 7]. We present an SVM classifier-based methodology which is an extended version of one-versus-the-rest multiclass SVM classifier. Leave-one-out cross-validation showed a high rate of accuracy (83% in total) for differentiating among the normal (normal healthy individuals), PAD, L4, and L5 groups.

(*3) Derivation of Medically Meaningful Differentiation Features Available for Visual Examination*. The key for obtaining differentiation with high accuracy is how to construct/select the features, that is, the input to classifiers. Our own previous reports [3, 7] show that different diseases produce kinematical differences in gait patterns. Focusing on this fact, we select/produce kinematical features in gait patterns for the differentiation. Additional advantage is that because these features are visually detectable and medically meaningful/consistent, these features are also available for a medical interview or visual examination.

*Related Works*. We have conducted the only studies thus far to use gait analysis to differentiate between patients with LSS and PAD [3, 7]. The present study differs from our past reports [3, 7] in its inclusion of the presented classification methodology and the results thereof as well as in the added differentiation features (amplitude of the femur angle, maximally contracted muscle length of the gastrocnemius muscle from the reference model, and maximally relaxed muscle length of the quadriceps muscle from the reference model). If we do not restrict ourselves to differentiation of the causes of intermittent claudication, there have been a number of studies concerning the analysis of intermittent claudication and the classification of gait patterns. Here, we briefly review them.

*Gait Analysis of Patients with LSS*. Suda et al. [8] evaluated the improvement in gait after surgical treatment of patients with neurogenic intermittent claudication. Papadakis et al. [9] compared the gait patterns of healthy people with those of patients with LSS; they also evaluated the postoperative progression of the gait pattern of patients with LSS [10] and showed that the variability of the gait decreased relatively to the preoperative gait pattern. Yokogawa et al. [11] compared gait patterns between patients with lumbar spinal canal stenosis (L4 radiculopathy) and those with osteoarthritis of the hip and found several differences.

*Gait Analysis of Patients with PAD*. Scherer et al. [12] compared the gait patterns of healthy people with those of patients with PAD and found several distinctive characteristics of the patients' walking gaits. Myers et al. [13] compared the gait patterns of patients with PAD before and after the onset of pain and found the gait to differ only at the ankle joint. Gardner et al. [14] compared the gait patterns of healthy people with those of patients with PAD and found differences in walking parameters such as the walking speed, stride length, and swing and stance times.

*Classification of Gait Patterns*. The gait analysis methods used for classification have been summarized previously [15, 16]. Wang et al. [17] presented a decision tree-based algorithm for classifying human walking motion/behavior. Kamruzzaman and Begg [18] identified and classified children with a cerebral palsy-related gait via an SVM-based method. Mezghani et al. [19] derived features and constructed a classifier for distinguishing asymptomatic from knee osteoarthritis-affected gait patterns.

## 2. Materials and Methods

Here, the methodology for obtaining gait pattern, the extracted features, and the SVM classifier-based methodology for the differentiation are described.

### 2.1. Methodology for Obtaining Gait Pattern

#### 2.1.1. Participants

The participants were 13 normal healthy individuals (5 men and 8 women), 10 patients with PAD (9 men and 1 woman), 13 patients with L5 LSS (4 men and 9 women), and 10 patients with L4 LSS (6 men and 4 women). The group to which each participant belonged was determined by his or her medical diagnosis, which was established by the medical doctors among the authors. The diagnoses were made from comprehensive consideration of the patients' clinical features; radiological findings; surgical findings; MRI, magnetic resonance angiography, ABI, and contrast-enhanced computed tomography results; and the effects of selective nerve root blocks. These experiments were approved by the Medical Ethics Committee of Koseiren Takaoka Hospital.

#### 2.1.2. Motion Capture

**Figure 1:**
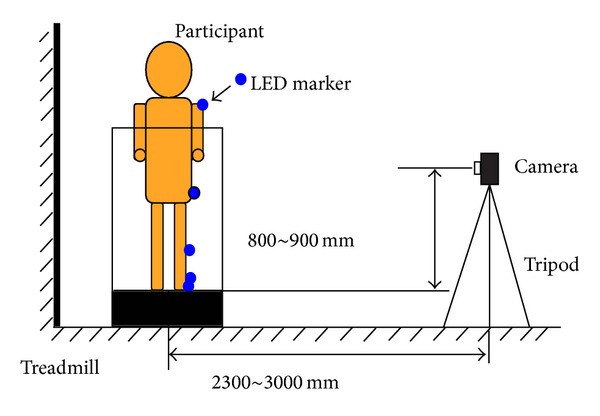
Walking measurement system.

Figure 1 shows the walking motion measurement system. As simplicity was one of our requirements, we constructed the system to obtain 2-dimensional gait patterns and aimed to derive the differentiation features and differentiate the diseases using the minimum amount of information. We constructed the measurement system as simply as possible, with easy set-up and calibration, to allow its use in small hospitals even by a few nonspecialized medical personnel. Other aims were a short duration of measurement, ability to obtain measurements in a narrow space, and ability to obtain measurements in a noncontrolled environment.

**Figure 2:**
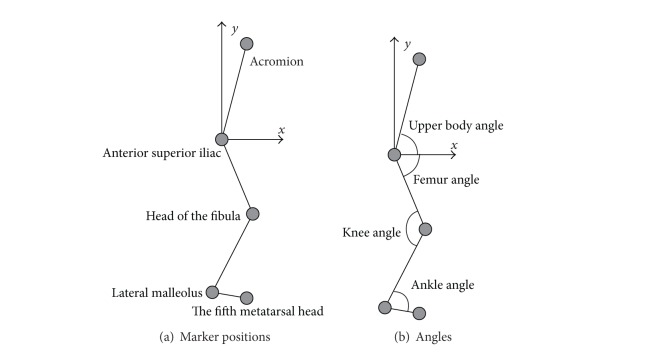
Coordinate frame, marker positions, and angles.

We measured the participants' gait patterns using light-emitting diode (LED) markers and had them walk on the treadmill in semidarkness so that the LED marker positions could be easily captured. Semidarkness can convert a noncontrolled environment into a controlled environment. We placed handmade LED markers on each participant's acromion, anterior superior iliac, fibular head, lateral malleolus, and fifth metatarsal head.  Figure 2(a) shows the definition of the coordinate frame and the marker positions. Note that the right side is forward in Figure 2. The LED markers were attached to the impaired leg. The participants practiced walking on the treadmill before the experiment for safety and to determine the appropriate treadmill speed. The latter was set to allow the participant to walk normally. The measurement was stopped if the participant felt pain. For safety, medical doctors stood by and watched each participant so that they could immediately stop the treadmill and help the participant in the event of an accident. We used a commercially available camera with a frame rate of 30 frames/s. Note that the obtained angle values were only used for analysis and that no calibration (such as measurement of leg length) was required or conducted.

#### 2.1.3. Analysis

The angles used for analysis are shown in Figure 2(b). We detected the marker positions using our own algorithm [3] based on an LK (Lucas-Kanade) filter [20]. We derived the angles from the marker positions. Note that the angles are not identical to the actual joint angles because they are mapped on the sagittal plane, and we therefore renamed them according to our own system. The mean data for 1 cycle of gait pattern were analyzed. The accuracy of this system depends on the resolution of the camera and the distance between the treadmill and camera and was from 0.007 to 0.04 [rad] for our set-up.

### 2.2. Extracted Features

Our goal was to identify the causative disease of intermittent claudication. As described previously, the candidate diseases are PAD and 2 varieties of LSS, L5 and L4. We also included a number of normal healthy participants as a control group. In order to get the features, that is, the input variables for classifiers, we extract the features of gait pattern in the 4 groups; normal, PAD, L5, and L4.  Table 1 shows the list of the extracted features, including the information about which features are used in the presented SVM classifier-based methodology described in Section 2.3. Focusing on the characteristic of each disease such as the areas where disruption of sensation and ischemia occur, we extracted the features associated with the motions of the angles of single joints. We remark about how to the knee angle at the start of the stance phase. The start of the stance phase was defined as the time at which the *x*  component of the marker attached to the fifth metatarsal head was maximal. The time at which the foot reaches its most forward position is not always identical to the start of the true stance phase, which it may precede. Therefore, we define that the knee angle at the start of the stance phase is the mean of the values of the knee angles from 4 frames before the start of the stance phase to the start of the stance phase. Letting *q*_3_(*k*) be the knee angle at *t* = *k* and *k*_ss_ be the time at which the stance phase started, we calculated the mean *q*_3_(*k*) from *t* = *k*_ss_ − 4 to *t* = *k*_ss_. This was recorded as the value of the knee angle at the start of the stance phase.

**Figure 3:**
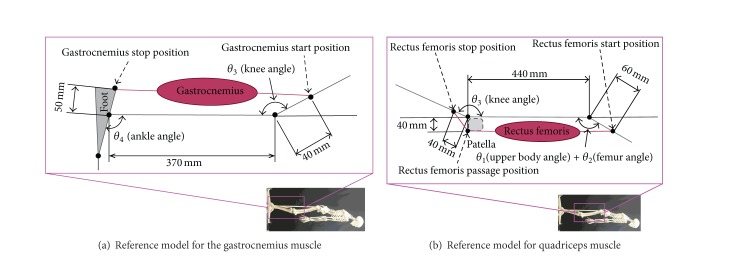
Reference models for calculating normalized biarticular muscle lengths.

The analysis of the angles of single joints corresponds to analysis of monoarticular muscles. However, there are also muscles that influence the angles of 2 adjacent joints, and it may be possible to derive features associated with these biarticular muscles. Therefore, we also extracted the features associated with biarticular muscles. Individual differences in body size make direct comparison of muscle lengths illogical; therefore, we used a reference model based on the bone lengths of the actual human skeleton model in our laboratory. We based the sites of attachment of the muscles to the bones in the reference model on anatomical data [21, 22].  Figure 3 shows the resulting reference model. We focused on the gastrocnemius muscle where it is considered to be the affected area for PAD, and the quadriceps muscle where it is considered to be the affected area for L4. The input for this model was the knee and ankle (upper body, femur, and knee) angles of a given participant and the output was the length of the gastrocnemius (quadriceps) muscle in the reference model. The value thus derived differed from the participant's actual muscle length but could be compared with those of the other participants. We therefore let the muscle length derived from the model represent the participant's muscle length. These values could thus be said to represent normalized muscle lengths.

### 2.3. SVM Classifier-Based Methodology for the Differentiation

#### 2.3.1. Methodology Based on the Support Vector Machine (SVM)

We will first introduce SVM [23–25]. SVM was originally a binary (2-class) classifier. Consider the given data set (**x**_1_, *y*_1_),…, (**x**_*m*_, *y*_*m*_), where **x**_*i*_ ∈ *R* ^*n*^ and *y*_*i*_ ∈ {−1, 1} is labeled for **x**_*i*_. Then, we solve the following quadratic function:

(1) min⁡w,b,ξ⁡12wTw+C∑i=1mξiSubject  to    yi(wTϕ(x)+b)≥1−ξi ξi≥0.

Here, the function *ϕ* maps data **x**_*i*_ to the higher-dimensional feature space *ϕ* : *R* ^*n*^ → *F*. The following hyperplane in the feature space splits the data into the 2 labeled classes:

(2) $$\begin{array}{c} f\left(x\right)=w^{T}\phi \left(x\right)+b, \end{array}$$

where **w** and *b* are the parameters that specify the linear hyperplane. This can provide a nonlinear boundary for the 2 labeled classes in the original data space. Note that the hyperplane containing *y*_*i*_(**w** ^*T*^ *ϕ*(**x**) + *b*) = 1 − *ξ*_*i*_ is called the support vector. *ξ*_*i*_ is termed the slack variable, and its solution gives the maximal margin for classification error. *C* > 0 is the parameter controlling the tradeoff between the number of misclassified data points in the training and the separation of the remaining data with the maximal margin. The function *ϕ* constructs a kernel function such as *K*(**x**_*i*_, **x**_*j*_) = *ϕ*(**x**_*i*_)^*T*^ *ϕ*(**x**_*i*_). The following are well-known candidates for the kernel function:

(3) K(xi,xj)=xiTxj linear,K(xi,xj)=(γxiTxj+r)d, γ>0 polynomial,K(xi,xj)=exp⁡(γ||xi−xj||2), γ>0 RBF,K(xi,xj)=tanh(γxiTxj+r) sigmoid,

where *γ*, *r*, and *d* are kernel parameters and RBF is the radial basis function. Given test data **x**_*t*_, the classifying decision is made by

(4) $$\begin{array}{c} \mathrm{sgn}\left(f\left(x_{t}\right)\right)=\;\mathrm{sgn}\left(w^{T}\phi \left(x_{t}\right)+b\right). \end{array}$$

The goal of our differentiation was to determine to which of the normal, PAD, L5, and L4 groups a given (test) dataset belongs. Therefore, a 4-class classifier was needed. The binary SVM classifier can be extended to a multiclass classifier [23, 25, 26]. There are 2 main approaches to constructing a *k*-class SVM. The first is to train the binary classifier with regard to all combinations (totaling *k*(*k* − 1)/2). If test data are given, we apply the *k*(*k* − 1)/2 classifiers to it and decide the final output by voting, with the most-voted class being the final output. This method is called one-versus-one (OVO). The other approach is to train a *k*-independent binary classifier by training the *i*th classifier to regard the *i*th class as the class with the positive label and the remaining classes as the class with the negative label. The *i*th classifier decision is made by

(5) $$\begin{array}{c} \mathrm{sgn}\left(f_{i}\left(x_{t}\right)\right)=\;\mathrm{sgn}\left(w_{i}^{T}\;\phi \left(x_{t}\right)+b_{i}\right). \end{array}$$

The overall decision is made by

(6) $$\begin{array}{c} i=\underset{i}{\text{argmax}}\;\;f_{i}\left(x_{t}\right). \end{array}$$

This approach is called one-versus-the-rest (OVR).

**Figure 4:**
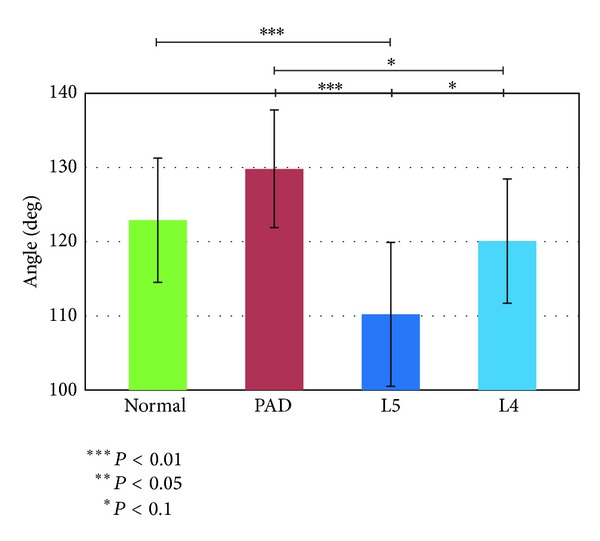
Mean ankle angle.

*Presented Methodology*. Not all features are always suitable for differentiating a certain group from the other groups (see the data for the features shown in Figures 4~12). In this case, a voting-based method, which does not take the magnitude of the possibility value into account (but instead provides 0 or 1), does not always work well. With this in mind, we present a differentiation methodology based on OVR. Other merits of OVR are that it is simple and first-running and that it is easy to optimize the parameters for the classifications. The methodology is as follows.

*Training*. We trained 4 binary classifiers, each of which was for classifying 1 class versus the other classes: (1) normal versus the other groups, (2) PAD versus the other groups, (3) L5 versus the other groups, and (4) L4 versus the other groups. We selected appropriate features for every binary classifier based on the significant differences described in Section 3. The features selected are shown in Table 1. In the table, “+” denotes the selected features and “−” the nonselected features for each classifier. The first row shows the target class for the binary classifier: for example, “normal” means the classifier for distinguishing the normal group from the other groups. 

*Evaluation*. First, the corresponding features of the given test/sample (walking motion) data were calculated. Using these features, we calculated the decision value *f*_*i*_(**x**_*t*_) (5) for each classifier. Then, from (6), we obtained the final output.

## 3. Results and Discussion

### 3.1. Features Associated with the Motions of the Angles of Single Joints

**Figure 5:**
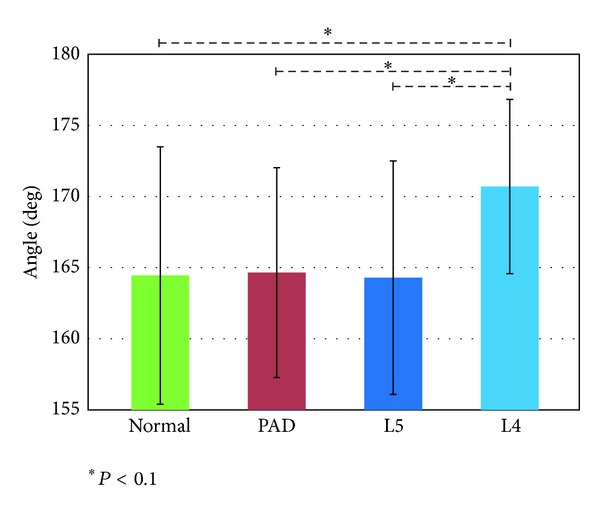
Knee angle at the start of the stance phase (the dashed lines show the results of* t*-tests conducted for each indicated pair).

**Figure 6:**
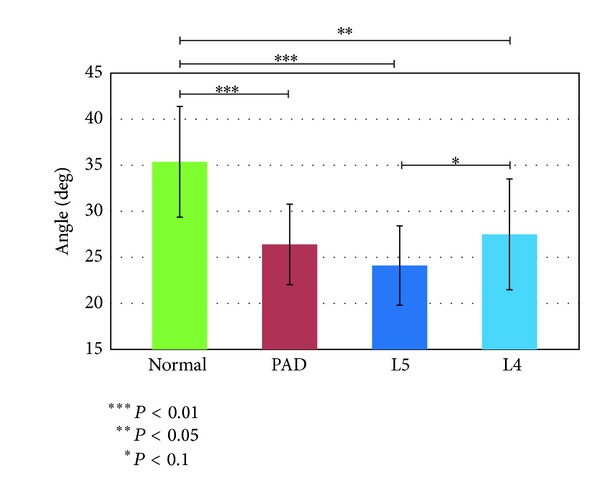
Amplitude of the femur angle.

We first investigated the features associated with the motions of the angles of single joints. Figures 4, 5, and 6 show the results.

#### 3.1.1. Mean Ankle Angle

Figure 4 shows that the ankle angle was larger in the PAD group and smaller in the L5 group than in the normal and L4 groups and differed significantly between the PAD and L5 groups according to the Tukey-Kramer method. A multiclass classification such as the SVM (OVR) generally requires classification/differentiation between a given group and all other groups, so the most useful features are those that are largest or smallest for a certain (target) group. Therefore, the mean ankle angle appeared useful for differentiating either PAD or L5 from the other groups.

Patients with PAD are susceptible to ischemia of the triceps surae muscles and therefore move to prevent the collapse and stenosis of the blood vessels inside these muscles. The patient attempts to keep the radii of the blood vessels large in order to minimize the loss of blood flow and thus avoid muscle ischemia. To accomplish this, the patient with PAD tends to keep the angle of the ankle large at all times. In contrast, patients with LSS (L5) have disruption of sensation around the tibialis anterior muscle and the bottom surface of the foot. This increases the risk for collision between the tip of the foot and the ground. In order to decrease this risk, patients with L5 tend not only to keep the ankle angle small but also not to lift up their legs, which makes their walking resemble shuffling. These trends are considered to produce large mean ankle angle in PAD group while small in L5 group.

#### 3.1.2. Knee Angle at the Start of the Stance Phase

Figure 5 shows that this feature is large in the L4 group, although the difference was not significant in Tukey-Kramer method; however, when we conducted* t* tests for each pair, we obtained significant differences between L4 and the other groups, shown in Figure 4 by the dashed lines. The difficulty of differentiating L4 from the other groups is evident; however, we anticipated that the knee angle at the start of the stance phase would be effective for differentiating the L4 group from the other groups despite the lack of a marked significant difference.

The L4 group experiences disruption of sensation around the quadriceps muscle, which causes large unexpected bending/flexion of the knee angle just after landing. In order to walk smoothly despite the unexpected bending/flexion, the patient tends to keep the quadriceps muscle contracted, especially during landing. The large knee angle at the start of the stance phase is consistent with this observation. The value was close to 180°, indicating that the knee is extended as much as possible. This configuration with the extended angle is a singular pose from the viewpoint of robotic manipulators [27] and enables the force along the links to be resisted without any additional joint torque, allowing absorption of the impulsive force in that direction upon landing. This may be one reason why the L4 group maintained a knee angle close to 180°.

#### 3.1.3. Amplitude of the Femur Angle

Figure 6 shows that the femur angle should be useful for differentiating the normal group from the other groups.

### 3.2. Features Associated with Biarticular Muscles

**Figure 7:**
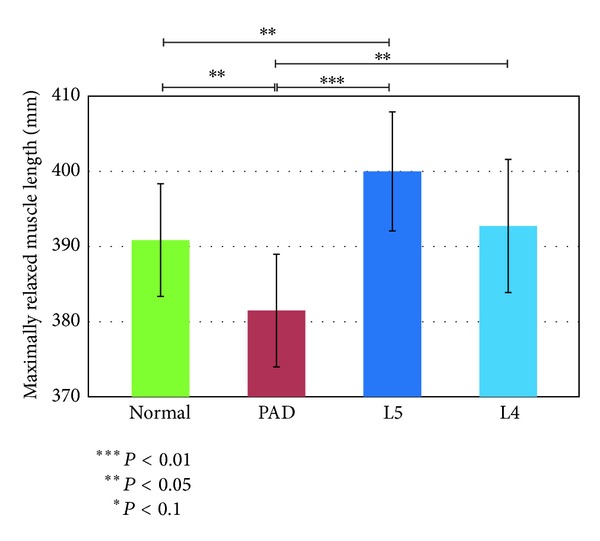
Maximally relaxed muscle length of the gastrocnemius muscle from the reference model.

**Figure 8:**
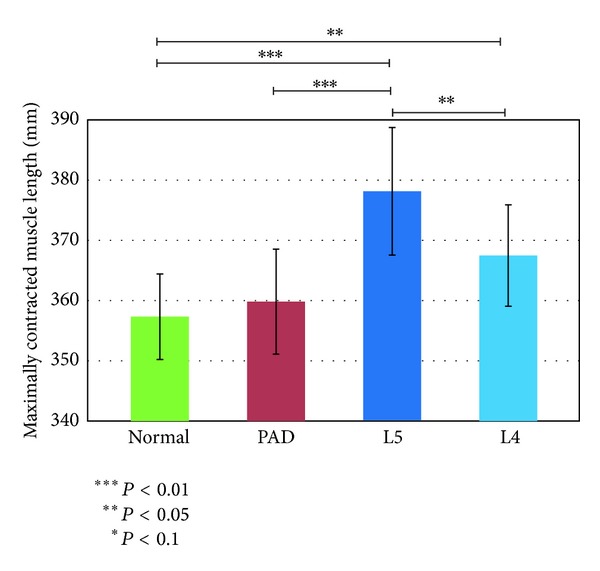
Maximally contracted muscle length of the gastrocnemius muscle from the reference model.

**Figure 9:**
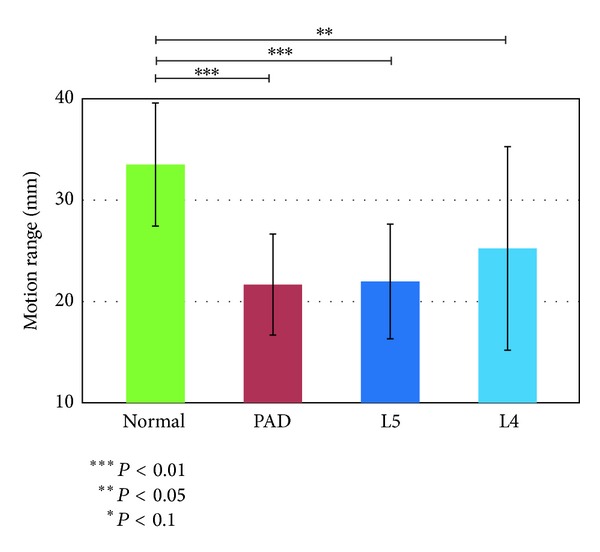
Range of motion of the gastrocnemius muscle from the reference model.

**Figure 10:**
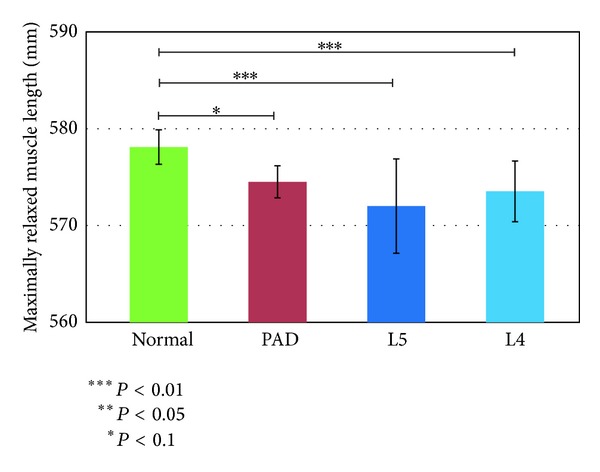
Maximally relaxed muscle length of the quadriceps muscle from the reference model.

**Figure 11:**
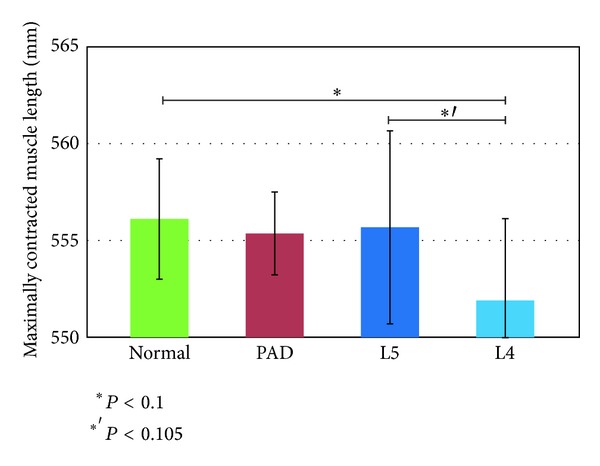
Maximally contracted muscle length of the quadriceps muscle from the reference model.

**Figure 12:**
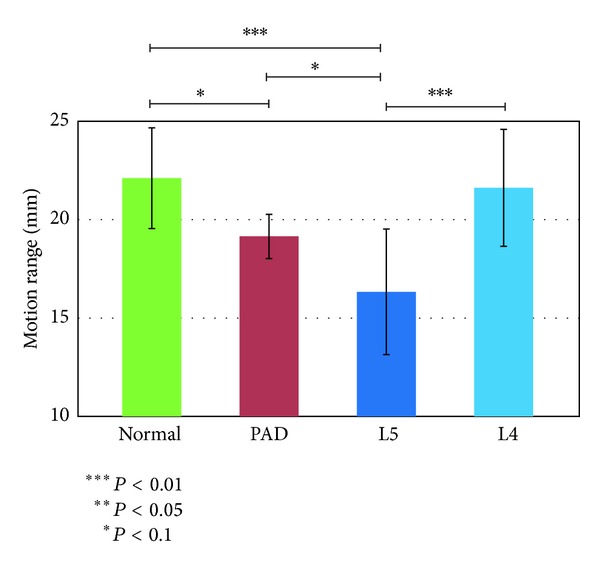
Range of motion of the quadriceps muscle from the reference model.

We used the models shown in Figure 3 and the angle data to calculate the maximum relaxed and contracted lengths of the muscles and their ranges of motion. Figures 7, 8, and 9 show the maximally relaxed and contracted lengths and the range of motion, respectively, of the gastrocnemius muscle from the reference model, and Figures 10, 11, and 12 show the same parameters for the quadriceps muscle from the reference model. We first considered the values for the length of the gastrocnemius muscle. Both the maximally relaxed and maximally contracted lengths increased from the PAD to the L4 to the L5 group. Only the normal group showed a different tendency, consistent with the results for the range of motion. Statistically significant differences were evident between many of the groups. The small values of every feature in the PAD group were consistent with the hypothesis that the patient keeps the length of the gastrocnemius muscle short. On the other hand, the large maximally relaxed and contracted muscle lengths and small range of motion in the LSS (L5) group support the hypothesis that the patient keeps the angle of the ankle small and avoids lifting up the legs. The maximally relaxed length appeared useful for differentiating PAD or L5 from the other groups, whereas the maximally contracted length appeared useful for differentiating L5 from the other groups. The range of motion was significantly greater in the normal group than in all other groups and would therefore be useful for differentiating the normal group from the other groups.

We next considered the values for the length of the quadriceps muscle. The maximally contracted length was smallest in the L4 group, although the differences were slight. This corresponds to the hypothesis that patients with L4 LSS keep the quadriceps muscle contracted, especially during landing. The range of motion was larger in the normal and L4 groups and smaller in the L5 group. This was attributed to the tendency of patients with L5 LSS to avoid lifting up their legs, as described above. This feature appeared potentially useful for differentiating between the L4 and normal groups and the other groups but more useful for differentiating the L5 group from the other groups. The maximally relaxed length was significantly larger only in the normal group and would therefore be useful for differentiating the normal group from the other groups.

### 3.3. Differentiation by Machine-Learning Based Methodology

Utilizing the extracted features listed in Table 1, we tried to differentiate the causative disease of intermittent claudication. For the comparison, we used not only the presented methodology described in Section 2.3 but also popular classifiers; LDA (linear discriminant analysis), decision tree, conventional one-versus-one SVM (OVO), and one versus-the-rest SVM (OVR). Matlab (Mathworks) statistics toolbox was used for LDA and decision tree, while implantation was conducted based on LIBSVM [24] for SVM. We used leave-one-out cross-validation for the evaluation. Note that we used normalized data for input to every classifier. We remark about the implantation of SVM. The implantation was conducted based on LIBSVM [24]. The RBF was chosen as the kernel function. The values for the 2 parameters *C* and *γ* for the classifications were determined by a grid search [24]. The grid search finds good values by evaluating (e.g., from cross-validation results) exponentially increasing values of *C* and *γ* (such as *C* = 2^−5^, 2^−3^, …, *γ* = 2^−5^, 2^−3^, …). Note that in the presented methodology, we applied the grid search to each classifier separately because the features used in each classifier were different.

Table 2 shows the classification results about total accuracy in every classifier.  Table 3 shows the detail of the classification. It can be seen that the presented method got very high accuracy, compared to the other methods. The total classification accuracy (83%) supported the efficiencies of the extracted differentiation features and the presented classification methodology.

The reason for low accuracy at conventional classifiers might be that a participant with a certain group does not always have the all extracted features in her/his gait pattern. This requires nonlinear classifier such as SVM. However, conventional SVM still did not show high accuracy. Not all features are always suitable for differentiating a certain group from the other groups. For example, the mean ankle angle shown in Figure 4 can easily differentiate the normal group from the PAD and L5 groups but cannot easily differentiate the normal group from the L4 group. This is the reason why we presented the new methodology.

If we focus the results of the presented methodology, aggregation of the classification results with respect to the group of test data yielded the highest classification accuracy for the PAD group and the lowest for the L4 group. The high classification accuracy for the PAD group is preferable from a clinical perspective because the failure to identify PAD can cause serious problems such as necrosis of the lower limbs. We attribute the high accuracy for this group to the large differences in the corresponding features between the PAD group and the other groups. In contrast, identification of the L4 group was difficult. This is true for the results in all the classifiers. One reason for this might be its similarity to the normal group; this might also be expected from Figures 4, 7, and 12. It can be seen from Table 3 that the presented methodology remarkably improved the accuracy for the identification of the L4 group. The identification of the L4 group is considered to be the key for the improvement of accuracy. The identification of other features for differentiating between the normal and L4 groups and further increasing the accuracy of the classification remains for future work.

## 4. Conclusion

Intermittent claudication is caused mainly by 2 different diseases, LSS (lumber spinal canal stenosis) and PAD (peripheral arterial disease). LSS can be subdivided into L4 and L5 disease. The medical treatments for these conditions are completely different, making their differentiation very important. At present, the diagnosis is made by analyzing many results from many examinations, including sophisticated methods such as MRI. This methodology is available only at well-equipped hospitals. With this in mind, this paper presents a novel SVM-based methodology for differentiating among normal healthy people and patients with PAD, L4, and L5 utilizing minimally required data, simple walking motion data. The simple walking measurement system was constructed to obtain 2-dimensional gait patterns in the sagittal plane and is intended for use at small hospitals or at home. The system's other key features are easy set-up and calibration, a short duration of measurement, usability in a narrow space by nonspecialists, and ability to obtain measurements in a noncontrolled environment. From the gait patterns, we then extracted several visually detectable and medically meaningful/consistent differentiation features that are also available for a medical interview or visual examination. The extracted features can largely be categorized into 2 groups: those associated with the angles of single joints (monoarticular muscles) and those associated with the angles of 2 adjacent joints (biarticular muscles). We used the derived differentiation features to construct an SVM- (support vector machine-) based differentiation method. The differentiation/classification was developed successfully and yielded a total accuracy of 83% in leave-one-out cross-validation. The accuracy of the differentiation/classification was lower for identification of patients with L4. Our future work will focus on improving the accuracy of this diagnostic method.

## Figures and Tables

**Table 1:** Extracted features for differentiating among normal, PAD, L5 and L4, and which features are used in the presented SVM classifier-based methodology. The first row shows the target class for the binary classifier: for example, “normal” means the classifier for distinguishing the normal group from the other groups. “+” denotes the selected features and “−” the nonselected features for each classifier.

Target group for classification	Normal	PAD	L5	L4
Mean ankle angle	+	+	+	+
Knee angle at the start of the stance phase	−	−	−	+
Amplitude of the femur angle	+	−	−	−
Maximally relaxed muscle length of the gastrocnemius muscle from the reference model	−	+	+	+
Maximally contracted muscle length of the gastrocnemius muscle from the reference model	+	+	+	+
Range of motion of the gastrocnemius muscle from the reference model	+	−	−	−
Maximally relaxed muscle length of the quadriceps muscle from the reference model	+	−	−	−
Maximally contracted muscle length of the quadriceps muscle from the reference model	−	−	−	+
Range of motion of the quadriceps muscle from the reference model	−	+	+	+

**Table 2:** Overall classification results.

Classifier	Accuracy [%]
LDA (linear discriminant analysis)	65
Decision tree	39
One-versus-one SVM (OVO)	70
One versus-the-rest SVM (OVR)	67
Presented SVM-based methodology	**83**

**Table:** (a) LDA (linear discriminant analysis)

Class of test data	Normal	PAD	L5	L4	All
Success	10	8	10	2	30
Failure	3	2	3	8	16

Total	13	10	13	10	46
Accuracy	0.77	0.8	0.77	0.2	0.65

**Table:** (b) Decision tree

Class of test data	Normal	PAD	L5	L4	All
Success	7	5	5	1	18
Failure	6	5	8	9	28

Total	13	10	13	10	46
Accuracy	0.54	0.5	0.38	0.1	0.39

**Table:** (c) One-versus-one SVM (OVO)

Class of test data	Normal	PAD	L5	L4	All
Success	12	8	9	3	32
Failure	1	2	4	7	14

Total	13	10	13	10	46
Accuracy	0.92	0.8	0.69	0.3	0.70

**Table:** (d) One versus-the-rest SVM (OVR)

Class of test data	Normal	PAD	L5	L4	All
Success	13	8	9	1	31
Failure	0	2	4	9	15

Total	13	10	13	10	46
Accuracy	1	0.8	0.69	0.1	0.67

**Table:** (e) Presented SVM-based methodology

Class of test data	Normal	PAD	L5	L4	All
Success	11	9	11	7	38
Failure	2	1	2	3	8

Total	13	10	13	10	46
Accuracy	0.85	0.9	0.85	0.7	0.83
